# The positive effect of physical constraints on consumer evaluations of service providers

**DOI:** 10.1371/journal.pone.0275348

**Published:** 2022-10-10

**Authors:** Yael Steinhart, Irit Nitzan, Jacob Goldenberg, David Mazursky

**Affiliations:** 1 Coller School of Management, Tel-Aviv University, Tel-Aviv, Israel; 2 Reichman University, Herzliya, Israel; 3 School of Business Administration, The Hebrew University, Jerusalem, Israel; University of Haifa, ISRAEL

## Abstract

Consumers tend to have negative perceptions of service providers that limit their freedom. People might therefore be expected to respond particularly negatively to service providers that physically limit their freedom of movement. Yet, we suggest that physical constraints that a service provider unapologetically imposes with no obvious logical justification (e.g., closing a door and restricting consumers to stay inside a room) may, in fact, boost consumers’ evaluations of the service provider. We propose that this effect occurs because consumers perceive such constraints as creating a structured environment, which they inherently value. Six studies lend converging support to these propositions, while ruling out alternative accounts (cognitive dissonance, self-attribution theory). We further show that the positive effect of physical constraints on evaluations is reversed when consumers perceive the constraints as excessively restrictive (rather than mild). These findings suggest that service providers may benefit from creating consumption conditions that mildly restrict consumers’ freedom of movement.

## 1. Introduction

It is widely believed that people tend to have negative perceptions of service providers that limit their freedom, and experience resentment toward and discomfort with those service providers [1]. In light of this tendency, one might expect consumers to respond particularly negatively to service providers that constrain them *physically*, that is, limit their preferred patterns of movement [2]. Indeed, the idea of being physically constrained elicits primarily negative connotations [3] and can even trigger psychological reactance [4].

The present research, however, suggests that physical constraints that a service provider imposes purposely and unapologetically—without providing clear logical justification for doing so—may boost consumers’ evaluations of the service provider. More importantly, we offer a theoretical understanding of why physical constraints might elicit such a positive effect.

Our proposition derives from both existing research and from actual consumption environments, which lend support to the idea that consumers do not always respond negatively when firms subject them to physical constraints. From the actual environment perspective, some popular firms constrain consumers’ movement as an inherent element of the consumption experience—e.g., IKEA stores, the Guggenheim Museum in New York City, and various historical and natural attractions (such as Antelope Canyon in Arizona), in which visitors’ movements are restricted to a pre-determined path. The success of these establishments suggests that consumers do not necessarily penalize firms that restrict their movements and may even reward them.

Of importance, our proposition stems from research that suggests that psychological reactance and negative connotations are not the only possible responses to restrictions on consumers’ freedom. Prior work [5,6], for example, has demonstrated that consumers may hold positive perceptions of restrictions on their freedom when these restrictions clearly contribute to the utility they gain from the service provider’s offering—e.g., by preventing undesired consequences, or by helping people attain expected utility from a service such as losing weight or saving more money. To our knowledge, however, no studies thus far have explicitly investigated whether *physical constraints* might positively affect consumer behaviors and attitudes toward the restricting service provider.

We propose that physical constraints may elicit positive responses because they enhance consumers’ sense that their environment is organized and structured—a state that people generally perceive as desirable [7]. The inherent desire for structure reflects a broader motivation to gain some degree of control over one’s surroundings [e.g., 8,9]. We further suggest that physical constraints imposed by a service provider affect consumer evaluations positively only when the constraints are mild, i.e., when they are not excessively intrusive or restrictive. When consumers experience an extreme level of physical constraint [3,9–11], we expect the effect to be reversed. We also rule out possible alternative accounts, including *dissonance reduction* [e.g., 12,13], which might suggest that positive effects of physical constraint may derive from activate rationalizations that justify their engagement with the service provider; and *self-attribution theory* [14], under which consumers may formulate their attitudes toward the service provider by observing their behavior and concluding that if they engaged in a constraining situation they must have liked it.

Our research sheds new light on how physical constraints affect attitudes and behavior in various contexts and, in doing so, highlights consumers’ basic desire to embrace structure and order [7–9,15]. Moreover, our findings point to practical benefits that service providers might derive from engaging with people in a manner that (mildly) restricts their movement. Specifically, the current research suggests that consumers might perceive physical constraints as a meaningful tangible signal of a structured environment, which service providers might leverage to make themselves more appealing.

In the next section, we elaborate on our rationale as to why physical constraints might be expected to positively influence consumers’ evaluations of service providers. We then formally present our hypotheses regarding the existence of this positive effect, its underlying mechanism, and our proposed moderator. We subsequently test our hypotheses in a series of experiments involving actual interactions between consumers and service providers, and rule out possible alternative accounts (cognitive dissonance and self-attribution). We conclude with a discussion of the theoretical and managerial implications of our findings and identify promising directions for future research.

## 2. Responses to physical constraints

Constraints imposed on individuals or firms may come in various forms, and the very existence of these limitations does not always indicate that the performance of those entities will be low compared to less constrained situations. For example, it was found that resource challenges can improve firms’ innovation performance [16], and that challenging conditions imposed on academic researchers, at the individual or team level, may enhance their effectiveness and innovativeness, overall improving their publications’ performance [17].

Physical constraints that restrict one’s movement have generally been shown to elicit negative psychological and even physiological responses. For example, in situations in which individuals are held physically captive against their will, captives tend to experience stress, negative feelings and anxiety—as in the case of prisoners of war [4,10,11] or individuals held hostage in crimes [18,19].

Additional studies have examined people’s responses to physical constraints imposed by conditions of confinement and isolation [2]. In most cases, confinement and isolation produce dramatic dysfunctions, including hallucinations and anxiety [20]. In some instances, however, some aspects of confinement and isolation may be perceived as relaxing, beneficial, and therapeutic, mainly due to the sensory deprivation that exists in such conditions [21,22].

A smaller stream of research has touched on the less extreme experience of physical constraint in consumption contexts, and specifically, feelings of confinement imposed by physical boundaries in a consumption space. One study [23], for example, explored the effect of ceiling height on consumers’ information processing, and showed that a low ceiling can prime confinement-related concepts and consequently prompt consumers’ use of predominantly item-specific processing (versus relational processing). Another study [24] examined how aisle width can evoke feelings of containment and enhance variety-seeking behaviors. These studies did not investigate constraints whose explicit purpose is to restrict consumers’ movements, as in the case of establishments that constrain their shoppers to a specific path or enclosed space or that require consumers to wear restrictive clothing such as safety harnesses or belts during consumption. Moreover, they did not explicitly consider how the presence of such constraints affects consumers’ evaluations of the service providers that impose them.

In this research, we address these aspects, predicting that constrained consumers may perceive the physical constraints that bind them—and the service provider imposing those constraints—in a positive way. We acknowledge that some physical constraints may provide consumers with direct utility, such as requirements to wear safety gear during physically risky activities; Indeed, in these cases it might seem obvious that consumers should embrace such constraints. A relevant research [6], for example, showed that consumers respond favorably to non-physical limitations on their freedom that provide clear utility. Rather, we focus on physical constraints that do not seem to be related to the primary engagement goal. We argue that consumers may perceive such physical constraints positively because they view them as a source of structure in their surrounding—and consumers tend to perceive structure as a valued utility. We discuss the rationale for this proposition in the following section.

We note that, in focusing on physical constraints, we do not address other types of restrictions that are common in the consumption context, such as specific dress codes [e.g., 25,26], reduced choice sets [e.g., 27–29], or a closed rather than open business ecosystem [e.g., 30], as in the case of Apple, whose products work well together but tend not to be compatible with third-party products [31]. Though the current research may be relevant to the latter types of restrictions to some degree, we focus on the unique effect of physically restricting consumers’ freedom of movement.

## 3. Desire for structure

Desire for structure can be broadly defined as individuals’ basic motivation to reduce the complexity that exists in the world and to gain some control over their environment [7,32] by seeking out simple structures and clarity [9,12]. More specifically, the tendency to seek out structure can serve to offset beliefs that people prefer to avoid, such as the belief that the outcomes in their lives are randomly determined, or that the world is governed by nothing more than chaos [8,9].

People may differ in their need for structure [9,12], and the desire for structure may be enhanced in certain situations. One study [7], for example, demonstrated that threatened consumers have a particularly strong tendency to seek structure in the consumption environment to regain a sense of control. These individual differences notwithstanding, people share a basic motivation to seek out structure in their environment. This motivation drives people in everyday life to establish routines that create structure, or to identify boundaries in the environment that define their experiences, such as a fence circling a house, or a simply organized environment [7,12]. In the consumption context, service providers can employ numerous strategies to enhance consumers’ perceptions of structure, such as having a prominent border surrounding a firm’s logo [7].

We suggest that, when consumers are subjected to physical constraints in various consumption contexts (assuming these constraints do not impose excessive inconvenience; See further discussion of this assumption in the following section), they are expected to benefit from the experience of these constraints. Specifically, consumers may perceive such constraints as a means of satisfying their desire for structure. Consumers may believe that the service provider has established constraints as well-defined boundaries whose purpose is to serve as an instrumental tool for enhancing consumers’ sense of structure. Therefore, when consumers encounter such physical constraints, they may perceive the service provider in a positive manner. Thus, the service provider also ultimately benefits from posing these constraints. In other words, consumers may believe that the service provider has established constraints as well-defined boundaries whose purpose is to serve as an instrumental tool for enhancing consumers’ sense of structure. Correspondingly, consumers may infer that the service provider is making an effort to reach out and to establish and maintain a structured, well-defined relationship with them. These perceptions might, in turn, enhance consumers’ likelihood of evaluating the service provider favorably.

Formally, we put forward the following hypotheses.

**H1:** Consumers who are physically constrained by a service provider will view the service provider more positively, compared with consumers who are not constrained.
**H2:** Sense of structure will mediate the positive effect of consumers’ physical constraints on service provider evaluations.

## 4. Will the positive effect of consumers’ physical constraints hold under extreme levels of constraints?

The hypotheses presented above include an inherent assumption that the physical constraints imposed by a service provider do not subject consumers to excessive discomfort. Prior research has suggested that individuals oppose restrictions in extreme cases, when they feel that these restrictions threaten their freedom—for example, when they are legally mandated to engage in specific behaviors [33], when they are prisoners of war [4,10,11] or when are being held hostages [18,19]. Drawing from these findings, we predict that when service providers impose physical constraints that are considered by consumers as extreme or overly restrictive of their freedom (e.g., by locking a door with a key instead of simply closing the door), consumers may no longer interpret this act as a means of structuring their environment but rather may perceive it as a means of controlling their behavior—and thus may evaluate the service provider less favorably. We formally hypothesize:

**H3:** The positive effect of physical constraints on consumers’ evaluations of a service provider will be undermined under extreme levels of constraints.

## 5. Alternative accounts

In testing our predictions, it is necessary to rule out alternative factors that might drive consumers’ tendency to express positive attitudes towards a service provider that constrains their freedom of movement. In what follows we outline potential alternative explanations and identify means of distinguishing them from perceptions of structured environment in the presence of physical constraints (the mechanism we propose).

Dissonance reduction is one mechanism that might drive consumers to express positive attitudes towards a service provider that constrains their movements [e.g., 12,13]. Specifically, physical constraints may cause consumers to experience discomfort and negative emotions. In order to avoid this adverse experience, consumers may activate rationalizations that justify their engagement with the service provider. Consequently, these rationalizations may generate positive evaluations of the service provider, similar to what we would observe in the presence of structured environment enhancement. However, we suggest that dissonance reduction and structured environment enhancement are likely to differ with regard to consumers’ perceptions of the physical constraints themselves. Specifically, under dissonance reduction, consumers are expected to perceive the experience of physical constraint negatively, whereas under structured environment enhancement we expect consumers to perceive the constraints positively (i.e., as a gateway to a more structured environment).

Another factor that might come into play when consumers engage with a service provider that physically constrains them is the mechanism of self-attribution [14]. According to self-attribution theory, people formulate their attitudes by observing their own behavior and concluding what attitudes must have caused it. Thus, consumers who choose to engage with a particular service provider may infer from this choice that they like the service provider, regardless of the physical constraints that the service provider imposes. In this case, we would not necessarily expect a service provider that imposes physical constraints to be perceived more favorably than a service provider that does not. As elaborated in what follows, the studies described below empirically rule out these alternative explanations.

## 6. Studies outline

We present the results of six studies, which provide converging evidence in support of our hypotheses regarding the positive effect of physical constraints on consumers’ evaluations of service providers that impose such constraints and of the underlying mechanism of this positive effect. The six studies manipulated physical constraints in different ways and in different contexts.

A set of tests demonstrated that the perceptions of the constrained consumption experiences in each study differ in the extent they were considered surprising and realistic, as well as in the degree to which it is possible to justify the service provider’s behavior. Specifically, we found that the constrained experiences in Studies 1 and 2 were surprising, unrealistic and difficult to be justified, while the constrained experiences in Studies 3–6 were considered highly realistic, easy to justify and not surprising (Full results of these tests can be found in the [S1 Study]). Notably, the tests consisted of descriptions of each constraint, without actually imposing them; while Studies 1 and 2 included actual experience. Nevertheless, assuming these constraints were only a small part of otherwise reasonable experiences, they are likely to stand out and impact consumers’ impression of the service provider. These results, therefore, not only highlight the counterintuitive nature of the positive effect of physical constraints but also suggest it takes place regardless of whether or not it matches consumers’ expectations or consumers’ ability to justify the constraints.

Studies 1 and 2 were designed to demonstrate the effect, supporting H1. Study 1 examined an actual interaction situation (i.e., receiving service from a hairdresser) in which some consumers were physically constrained (i.e., physically tied to the hairdresser’s chair), whereas others were not. In Study 2, which examined students’ engagement with an experimenter at their university, we subtly activated physical constraints by having the experimenter close the door of the lab. In Studies 3 and 4 we focused on the underlying mechanism of the effect, demonstrating that the positive effect of physical constraints is driven by consumers’ sense of structure (H2). Furthermore, Study 3 explicitly tested and confirmed our proposition that the effect emerges not only when physical constraints contribute to one’s primary goal but also when they are unrelated to this goal. In other words, this study enabled us to capture the distinctive favorable perception associated with physical constraint. Study 5 ruled out dissonance reduction and self-perception theory as mechanisms for the effect. Finally, in Study 6, we showed that the positive effect of physical constraints only takes place under mild levels of physical constraints; When constraints are extreme, the effect is reversed (H3).

In all studies, we used a standardized manipulation check measure for our physical constraints, by relying on existing research of physical constraints in the context of captivity [e.g. 3,10,11]. Specifically, we asked participants to rate the extent to which they felt themselves to be captives of the service provider. In Studies 1–5, in which we exposed some participants to mild physical constraints, we predicted that participants subjected to these constraints would rate their sense of captivity as low to mild (i.e., not exceeding mid-scale), but higher than participants who were not subjected to such constraints. In Study 6, we intentionally induced a situation with extreme constraints and expected participants to rate their sense of captivity in this condition above mid-scale.

The set of studies was approved by the ethic committee of first and second authors University. Participants viewed an approved information sheet before giving informed consent to take part.

## 7. Study 1: The positive effect of physical constraints on consumers’ evaluations

Study 1 lends support to H1, showing that physical constraints on the movements of consumers who are receiving a service can positively affect their evaluations of that service. This study took place in an actual service context, i.e., when receiving a haircut (or blow-dry). To carry out this study, we recruited two professional hairdressers, who came to the university and invited students to receive a haircut/blow-dry for an attractive price (students were asked to pay only $5 USD). We manipulated physical constraints by tying students to the hairdresser’s chair.

### 7.1 Method

**Fig 1:**
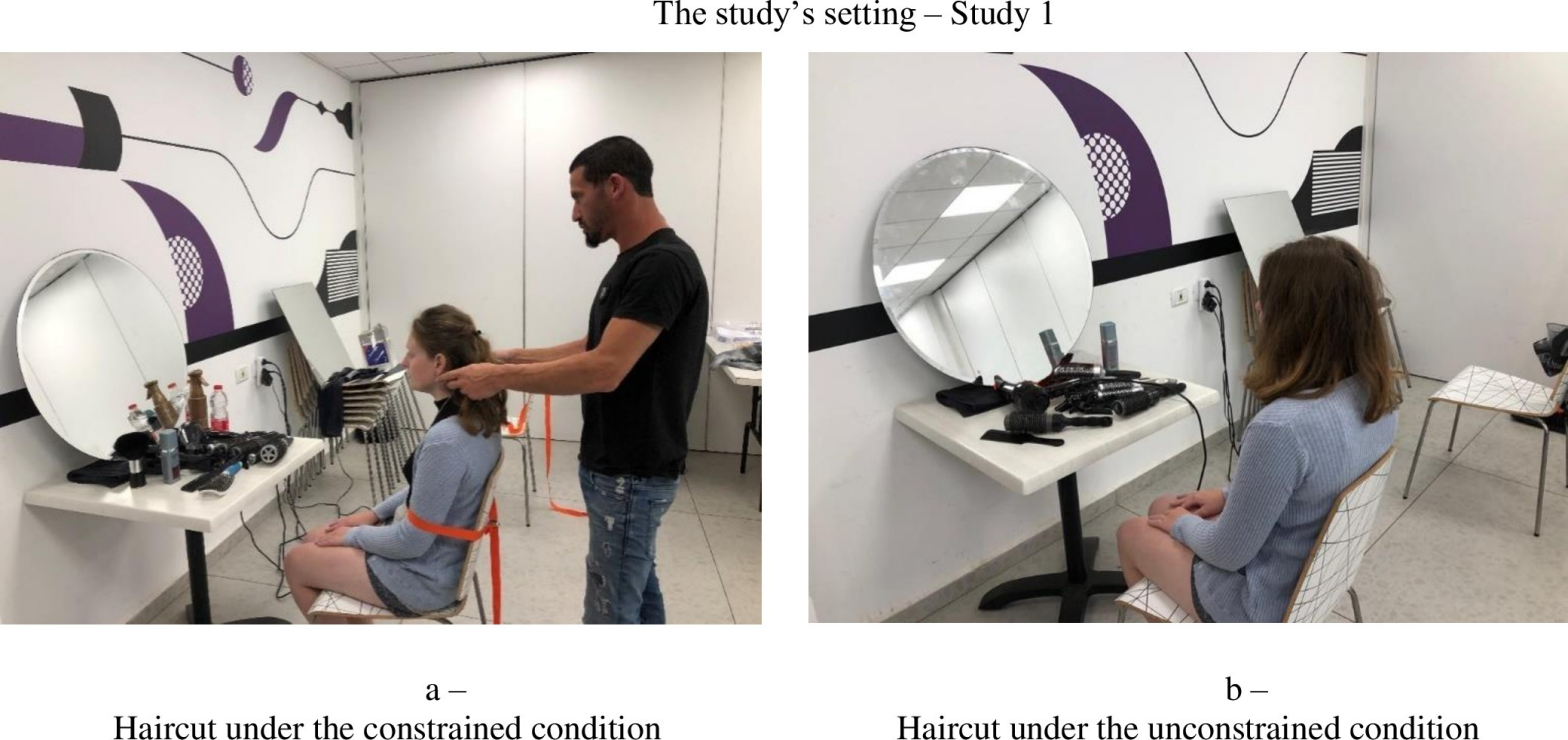
The study’s setting–Study 1. a. Haircut under the constrained condition. b. Haircut under the unconstrained condition.

Fifty-six participants (52% women, *M*_age_ = 27) participated in this study. Each participant was given a choice of receiving a haircut or a blow-dry. Participants were each randomly assigned to one of two conditions: constrained or unconstrained. In the constrained condition, the hairdresser used a belt to tie the participant to the chair and told him or her that this was how they were going to receive their haircut/blow-dry, providing no further explanation (see Fig 1A). In the unconstrained condition, participants sat freely in the chair and received their haircut/blow-dry (see Fig 1B).

None of the participants in either condition was aware of the other condition (e.g., participants in the unconstrained condition were not aware of the physically constrained condition and did not see the belt used in that condition). Moreover, each participant received his/her haircut/blow-dry in the absence of other participants in the room.

After receiving the service, participants completed a short questionnaire and paid $5 for the service. In the questionnaire, participants were asked to rate the extent to which the haircut was appealing and the likelihood that they would recommend the service to others, on a scale from 1 (*not at all*) to 7 (*very much*). Participants also rated the degree to which they felt they were the hairdresser’s captives, on a scale from 1 (*not at all*) to 7 (*very much*). A full description of the study can be found in the (S2 Study).

### 7.2 Results and discussion

#### Manipulation check of physical constraints

As expected, we found that participants in the constrained condition rated their perceived captivity higher (*M* = 3.03, *SD* = 1.91) than did participants in the unconstrained condition (*M* = 1.96, *SD* = 1.67, *F*(1, 54) = 4.74, *p* = .03; η^*2*^ = .08). Notably, participants in the constrained condition rated their captivity levels between low and mild, i.e., not exceeding the mid-scale, suggesting that their evaluations of the service were not likely to have been affected by perceptions of extreme constraints.

#### Main analysis

Since men and women differed in their service requests (e.g., all men requested a haircut while most women requested a blow-dry, with only a few requesting a haircut), we controlled for gender in our analysis. We found that participants in the constrained condition rated the haircut as more appealing (*M* = 6.54, *SE* = .11) and were more likely to recommend the service to others (*M* = 6.84, *SE* = .09) compared with participants in the unconstrained condition (*M*_*appealing*_ = 6.23, *SE*_*appealing*_ = .13, *F*(1, 53) = 3.12, *p* = .083; η^2^ = .06; *M*_*recommend*_ = 6.53, *SE*_*recommend*_ = .12, *F*(1, 53) = 3.93, *p* = .053; η^2^ = .07). Gender also had a significant effect on both ratings (appealing: *F*(1, 53) = 3.92, *p* = .053; η^2^ = .07; recommend: *F*(1, 53) = 5.33, *p* = .025; η^2^ = .09).

The results of this experiment provide initial support for H1 by indicating that in an actual service context, consumers who experienced mild physical restrictions perceived the provider or service more favorably than consumers who experienced no physical restrictions, as reflected in their service ratings and their willingness to recommend it to others.

## 8. Study 2: The positive effect of physical constraints on consumers’ choices

In Study 2, we aimed to lend robustness to the findings of Study 1 by replicating the positive effects of physical constraints (i) in a different setting, (ii) using a different dependent variable (actual choices as opposed to perceptions of the provider as an indication of participants’ evaluations), and (iii) using a different type of physical constraint—a closed- vs. open-door manipulation performed by the experimenter. Specifically, in this study, we relied on the fact that students in certain fields are required by their university to participate in academic experiments for credit, and thus we used our behavioral lab as a natural consumption setting for these students. As in Study 1, we pretested this study’s setting to ensure that the physical constraints imposed by the provider (the act of closing a door) did not seem to be related to the primary goal of the engagement experience.

### 8.1 Method

One hundred fifty-four undergraduate participants (47% women, *M*_age_ = 23.21) were invited to the lab to participate in an experiment for course credit. All participants were first-year students.

Participants were each randomly assigned to one of three conditions: closed door, open door, and control. In the closed-door condition, the experimenter told the participants that the experiment required that the lab door remain closed during the experiment, and that participants would not be permitted to leave the lab. She then closed the door and sat near the closed door. In the open-door condition, the experimenter told participants that the lab door would remain open during the experiment and that participants were permitted to leave the lab as they wished. She then opened the door and sat at a distance from the door. In the control condition, the door of the lab was closed as it usually would be during a lab experiment. The experimenter made no reference to the door in her instructions, and sat in her regular place (near the experimenter’s table).

Next, participants completed a filler task of jumbled brands (there was no significant difference in performance between conditions (*F*(1, 154) = 1.757, *p* = .176; η^*2*^ = .023). We then measured participants’ willingness to re-engage with the behavioral lab as our dependent measure. Specifically, all participants were told about an experimental panel that the behavioral lab researchers use when conducting their studies, whose members receive payment for their participation in experiments (in contrast to the current participants, who were receiving course credit). Participants were told that the researchers who manage this panel were recruiting new panel members. Participants were requested to state whether they would like to take part in the paid panel. Finally, participants rated the degree to which they felt they were captives on a scale from 1(*not at all*) to 7 (*very much*). A full description of the study can be found in the (S3 Study).

### 8.2 Results and discussion

#### Manipulation check of physical constraints

As expected, we found a significant effect of experimental condition on perceived captivity (*F*(2, 151) = 8.77, *p* < .001; η^*2*^ = .104). Specifically, participants in the closed-door condition rated their sense of captivity higher (*M* = 2.09, *SD* = 1.58) than did participants in the open-door condition (*M* = 1.31, *SD* = .62, *p* < .001) or in the control condition (*M* = 1.33, *SD* = .77, *p* < .001). The difference in reported sense of captivity between the open-door and the control conditions was not significant (*p =* .90). As in Study 1, perceived captivity was low (the average rating did not exceed mid-scale).

#### Main analysis

We conducted a chi-square test to examine whether participants’ desire to join the paid panel (0 = not join, 1 = join) was influenced by their experimental condition (0 = control, 1 = open door, 2 = closed door). In line with H1, this analysis revealed significant differences in choice patterns across experimental conditions (χ^2^(2) = 6.11, *p* = .047). Participants in the closed-door condition were more willing to join the paid panel (38.6%) than were participants in the open-door condition (28.8%, χ^2^(1) = 6.24, *p* = .012) or in the control condition (32.6%, χ^2^(1) = 2.87, *p* = .090). The latter difference was only marginally significant. Notably, the difference in participants’ willingness to join the paid panel between the open-door and control conditions was not significant (*p* = .389).

The results of this experiment suggest that participants responded positively to physical constraints that restricted their freedom of movement (H1), as reflected in their willingness to further participate in experiments in the behavioral lab in the future. In addition, the findings of Study 2, similarly to those of Study 1, lend support to our proposition that even constraints with no direct relation to the primary engagement goal may induce positive attitudes. In the next study, we delve further into this idea by explicitly comparing between the effects of physical constraints with versus without direct relation to the engagement experience. Additionally, we shed light on the underlying mechanism that elevates consumers’ evaluations in the presence of physical constraints.

## 9. Study 3: The mediating role of sense of structure

Study 3 had two main goals. The first was to lend further support to H1 by replicating the positive effect of physical constraints on individual evaluations, in a different setting. The second was to test our hypotheses regarding the underlying mechanism of the effect (H2). That is, we tested our proposition that physical constraints may signal to consumers that a service provider is inclined to create structure, order, and clarity in the way its service is delivered. Accordingly, in this study we presented participants with two situations involving physical constraints: one in which the constraint did not facilitate achievement of the engagement goal (constrained with long path); and another in which the constraint provided a direct benefit for consumers in terms of achieving the engagement goal (constrained with short path). We aimed to show that, compared with consumers who engage with a service provider without any physical constraints, those who are subjected to such constraints perceive the service provider as enhancing their sense of structure and perceive the service provider more favorably—both when they perceive the constraint as helping them exit the parking lot using a faster route and, more importantly, even when they acknowledged that the constraint causes them to exit the parking lot using a longer route.

### 9.1 Method

We recruited 256 undergraduate participants (52% women, *M*_age_ = 23.58), who took part in this experiment for course credit. Participants were each randomly assigned to one of three conditions in a between-subjects design: unconstrained, constrained with a short path, and constrained with a long path.

**Fig 2:**
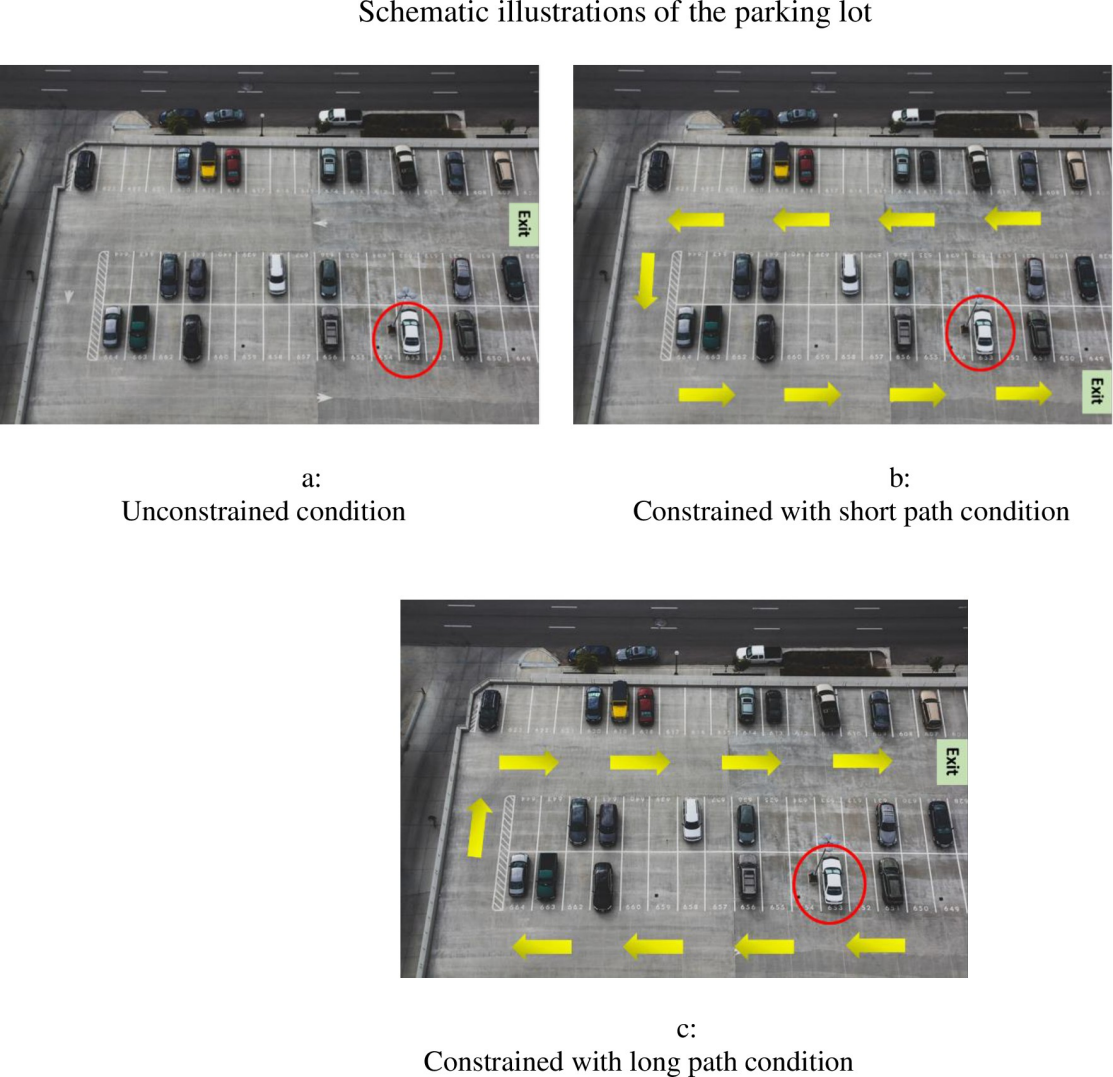
Schematic illustrations of the parking lot (Study 3). a. Unconstrained condition. b. Constrained with short path condition. c. Constrained with long path condition.

All participants were asked to imagine that they had just finished a session at the gym and were about to leave the parking lot where they parked their car. Then, participants were presented with a schematic photo of the parking lot featuring their specific parking space, marked as shown in Fig 2A, 2B and 2C (for the unconstrained, constrained with a short path, and constrained with a long path conditions, respectively). In the unconstrained condition, we asked participants to imagine driving through the parking lot in any direction they desired, including through empty parking spaces (as schematically presented in Fig 2A). In the ‘constrained with a short path’ condition, we asked participants to imagine that the parking lot’s management had marked the permitted directional flow on the parking lot, and that driving in the opposite direction or through empty parking spaces was not permitted. Specifically, participants were told that, based on their marked parking spot, their way out of the parking lot was the shortest possible path (as schematically presented in Fig 2B). Finally, in the ‘constrained with a long path’ condition, we presented a similar scenario to that of the constrained with a short path condition. However, we marked the flow of the arrows in the parking lot in the opposite direction (as schematically presented in Fig 2C), such that the participant’s path out of the parking lot was much longer than their path in the ‘constrained with a short path’ condition.

Participants were then asked to rate their overall evaluation of the parking lot management on a 7-point scale from 1 (*low evaluation*) to 7 (*high evaluation*). As a process measure, participants were asked to indicate the extent to which they believe the parking lot management establishes a structured experience, on a 7-point scale from 1 (*not at all*) to 7 (*very much*).

Finally, as a manipulation check of the experimental conditions, participants rated the degree to which they felt they were captives on a scale from 1 (*very little*) to 7 (*very much*). As a manipulation check, we also asked participants to rate, on a scale from 1 (*not at all*) to 7 (*very much*), the extent to which the policy of the parking lot management facilitates the way out of the parking lot. A full description of the study can be found in the (S4 Study).

### 9.2 Results and discussion

#### Manipulation check of physical constraints

As expected, we found a significant effect of experimental condition on perceived captivity (*F*(2, 253) = 14.19, *p* < .001; η^*2*^ = .10). Specifically, least significant difference (LSD) post-hoc comparisons showed that participants in the ‘constrained with a long path’ condition rated their sense of captivity higher (*M* = 2.99, *SD* = 1.78) than did participants in the unconstrained condition (*M* = 1.77, *SD* = 1.41, *p* < .001). Similarly, participants in the ‘constrained with a short path’ condition (*M* = 2.10, *SD* = 1.45) rated their sense of captivity higher than did those in the unconstrained condition (*p* < .001). The difference in perceived captivity between the two constrained conditions was not significant (*p* = .178). As in Studies 1 and 2, the average rating of perceived captivity did not exceed mid-scale.

#### Manipulation check of facilitating the way out of the parking lot

As expected, an ANOVA revealed a significant effect of experimental condition on participants’ perceptions of the extent to which the parking lot management policy facilitates the way out of the parking lot (*F*(2, 253) = 40.65, *p* < .001; η^*2*^ = .243). Specifically, post-hoc analysis (LSD) revealed that participants in the ‘constrained with a long path’ condition assigned lower ratings to this item (*M* = 2.97, *SD* = 1.64) than did participants in the unconstrained condition (*M* = 5.15, *SD* = 1.59, *p* < .001) or in the ‘constrained with a short path’ condition (*M* = 4.67, *SD* = 1.77, *p* < .001). The difference between the two constrained conditions was marginally significant (*p* = .060).

#### Main analysis

An ANOVA of participants’ overall evaluation of the parking lot management revealed a significant effect of experimental condition (*F*(2, 253) = 3.76, *p* = .025; η^*2*^ = .029). Specifically, consistent with H1, post-hoc analysis (LSD) revealed that participants in the ‘constrained with a long path’ condition gave higher ratings to the parking lot management (*M* = 4.73, *SD* = 1.48) compared with participants in the unconstrained condition (*M* = 4.29, *SD* = 1.37, *p* = .034). Similarly, participants in the ‘constrained with a short path’ condition gave higher ratings to the parking lot management (*M* = 4.82, *SD* = 1.20) than did those in the unconstrained condition (*p* = .011). Consistent with participants’ captivity perceptions, the difference in evaluations of parking lot management between the two constrained conditions was not significant (*p* = .67).

A separate ANOVA of participants’ sense of structure scores revealed a significant effect of experimental condition (*F*(2, 253) = 75.44, *p* < .001; η^*2*^ = .374). Specifically, in line with our prediction, post-hoc analysis (LSD) revealed that participants in the ‘constrained with a long path’ condition reported a greater sense of structure (*M* = 5.57, *SD* = 1.32) compared with participants assigned to the unconstrained condition (*M* = 3.20, *SD* = 1.57, *p* < .001). Similarly, participants in the ‘constrained with a short path’ condition reported a greater sense of structure (*M* = 5.44, *SD* = 1.375) than did those in the unconstrained condition (*p* < .001). The difference in sense of structure between the two constrained conditions was not significant (*p* = .55).

#### Mediation analyses

To check whether *sense of structure* mediated the effect of experimental condition on participants’ evaluations of the parking lot management, we conducted a mediation analysis following a bootstrapping procedure (Model 4, [34]). Specifically, we regressed participants’ evaluations on experimental condition as the predictor (dummy variable X1: 1 = constrained with a long path; 0 = unconstrained, constrained with a short path, dummy variable X2: 1 = constrained with a short path, 0 = unconstrained, constrained with a long path), with the sense of structure score (mean-centered, *M* = 4.73) as the mediator. The analysis confirmed H2, which predicted that the effect of experimental condition on participants’ evaluations would be fully mediated by sense of structure when comparing the ‘constrained with a long path’ condition and the unconstrained condition (X1: *b* = 1.13, *SE* = .16, 95%, CI: .837 to 1.48). We also found a significant mediation effect when comparing the ‘constrained with a short path’ condition and the unconstrained condition (X2: *b* = 1.07, *SE* = .157, 95%, CI: .787 to 1.393).

The results of Study 3 lend further support to H1, suggesting that participants who were subjected to physical constraints perceived the service provider (parking lot management) more favorably compared with participants whose movements were not constrained (unconstrained condition). Our analyses further demonstrated that the experience of mildly restrictive physical constraints elevated consumers’ sense of structure, which, in turn, drove their positive evaluations of the service provider (H2). Of importance, these results suggest that the presence of physical constraints can enhance consumers’ evaluations of the service provider, regardless of the extent to which such constraints are perceived as directly enhancing the service consumption experience.

## 10. Study 4: Process via moderation: Manipulating sense of structure

Study 4 aimed to lend further support to our proposed underlying mechanism—sense of structure—by manipulating the extent to which participants generally attribute positive versus negative value to structure in life. We expected the presence of physical constraints (versus a lack of constraints) to positively affect evaluations by consumers who were primed to generally perceive structure positively. Conversely, we expected the effect to be attenuated among participants primed to perceive order and structure in life in a negative manner. We used a scenario similar to that presented in Study 3. Since in Study 3 both constrained conditions yielded similar results, in this study we compared a single constrained condition to an unconstrained consumption experience (with no reference to the benefits of the physical constraint).

### 10.1 Method

We recruited 280 participants (46% women, *M*_age_ = 35.02) to take part in an online study for which they received $1 USD. Participants were each randomly assigned to one of four conditions in a 2(structure perception: positive vs. negative) × 2(physical constraints: unconstrained vs. constrained) between-subjects design.

First, we manipulated structure perceptions. Participants in the positive-structure-perception condition were asked to think of situations in which order and structure had helped them to cope, and to give a specific example of such a situation. Participants in the negative-structure-perception condition were asked to write about an example in which order and structure had distracted them from coping effectively in a specific situation.

**Fig 3:**
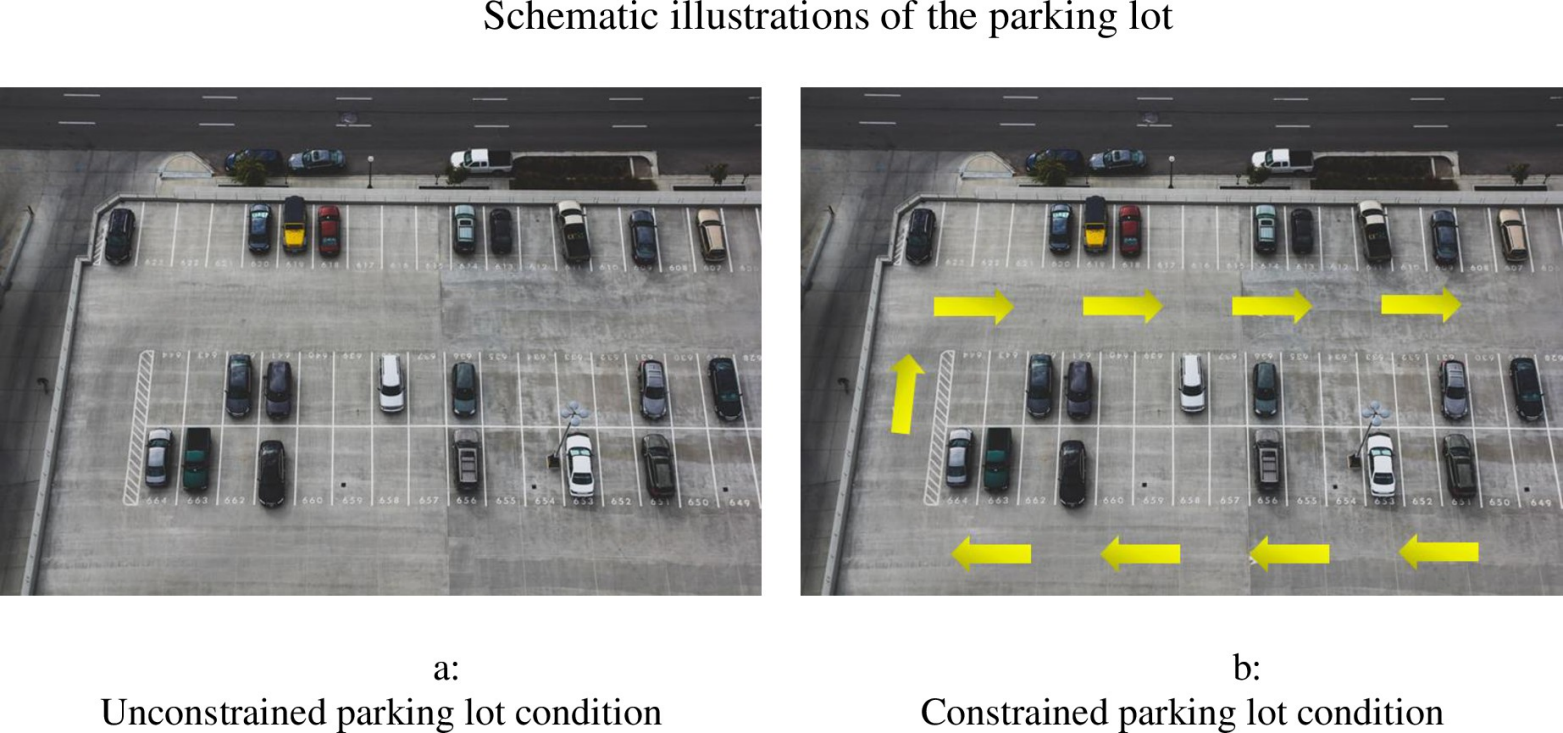
Schematic illustrations of the parking lot. a. Unconstrained parking lot condition. b. Constrained parking lot condition.

Then, we presented participants with a parking lot scenario similar to that used in Study 3, accompanied by a schematic photo of the parking lot (Fig 3). In this study, the specific location of the participant’s car was not mentioned, and its location was not indicated in the photo. Participants in the unconstrained condition were asked to imagine going to their usual parking lot and leaving it by driving through it in any direction they desired, including through empty parking spaces (as schematically presented in Fig 3A). Participants in the constrained condition were asked to imagine that the parking lot’s management had marked the permitted directional flow on the parking lot, and that driving in the opposite direction or through empty spaces was not permitted (as schematically presented in Fig 3B).

As in Study 3, participants were asked to rate their overall evaluation of the parking lot management on a 7-point scale from 1 (*low*) to 7 (*high*). As a manipulation check measure of structure perceptions, they were also asked to rate their agreement with the following statement: “I am in favor of having order and structure in my life” on a 7-point scale from 1 (*strongly disagree*) to 7 (*strongly agree*). As a manipulation check of physical constraints, as in Study 3, participants rated their sense of captivity on a scale from 1 (*not at all*) to 7 (*very much*). A full description of the study can be found in the (S5 Study).

### 10.2 Results and discussion

#### Manipulation check of structure perceptions

As expected, we found a significant effect of structure perception condition on participants’ perceptions of the extent to which structure in life is favorable (*F*(1, 278) = 4.12, *p* =. 043; η^*2*^ = .015). Specifically, participants in the positive-structure-perception condition indicated more favorable perceptions of structure in life (*M* = 5.75, *SD* = 1.11) than did participants in the negative-structure-perception condition (*M* = 5.46, *SD* = 1.27). Interestingly, in both conditions, ratings were above mid-scale, implying that participants generally preferred order and structure in life.

#### Manipulation check of physical constraints

As expected, we found a significant effect of physical-constraint condition on perceived captivity (*F*(1, 276) = 4.61, *p* = .033; η^*2*^ = .016). Specifically, participants in the constrained condition rated their sense of captivity higher (*M* = 2.97, *SD* = 1.99) than did participants in the unconstrained condition (*M* = 2.50, *SD* = 1.62). As in prior studies, in both cases, the average rating of perceived captivity was lower than mid-scale. Of importance, captivity perceptions were not significantly affected by structure-perception condition (*F*(1, 276) = .49, *p* = .50; η^*2*^ = .002) or by the interaction between structure-perception condition and physical-constraint condition (*F*(1, 276) = 1.18, *p* = .28; η^*2*^ = .004).

#### Main analysis

We conducted an ANOVA of participants’ overall evaluations of the parking lot management as a function of structure-perception condition and physical-constraint condition. The analysis revealed a significant effect of physical-constraint condition (*F*(1, 276) = 39.74, *p* < .001; η^*2*^ = .13). Specifically, as predicted in H1, participants in the constrained condition reported higher evaluations (*M* = 5.22, *SD* = 1.30) than did participants assigned to the unconstrained condition (*M* = 4.10, *SD* = 1.62). Of importance, the two-way interaction between structure-perception condition and physical-constraint condition was also significant (*F*(1, 276) = 4.51, *p* = .035; η^*2*^ = .016). When decomposing the interaction, we found that participants in the constrained condition rated the parking lot management more favorably than did participants in the unconstrained condition—for both the positive-structure-perception condition (*M*_constrained_ = 5.34, *SD* = 1.29; *M*_unconstrained_ = 3.87, *SD* = 1.63, *F*(1, 276) = 35.98, *p* < .001) and the negative-structure-perception condition (*M*_constrained_ = 5.10, *SD* = 1.31; *M*_unconstrained_ = 4.37, *SD* = 1.57, *F*(1, 276) = 8.63, *p* = .003). However, the slope of the relationship between physical-constraint condition and evaluations was significantly steeper for those who were primed to think positively of order and structure in life than for those who were primed to think negatively of order and structure in life (Z = 6.06, *p* < .001).

The results of Study 4 lend further support to the positive relationship between physical constraints and individual evaluations (H1), as well as to the proposed underlying mechanism of structure perceptions (H2). Specifically, we demonstrated that the positive effect of physical constraints is more pronounced when people are primed to think of the benefits (versus the disadvantages) of order and structure in life.

## 11. Study 5: Ruling out alternative accounts

Study 5 aimed to investigate cognitive dissonance reduction and self-attribution theory as alternative accounts for the positive effect of physical constraints on consumers’ evaluations. This study, which relied on a constraint manipulation similar to that described in Study 4, was conducted during the COVID-19 pandemic, when people were experiencing uncertainty in their lives. We embraced this situation and asked participants to share how the pandemic had affected their lives. We expected that such sharing would enhance participants’ desire for structured consumption, as such structure can provide a sense of control in the presence of uncertainty [8,9].

### 11.1 Method

We recruited 282 participants (55% women, *M*_age_ = 35.88) to participate in an online study for which they received $1 USD. Participants were each randomly assigned to one of two conditions: unconstrained and constrained. First, all participants were asked to think of their current situation amid COVID-19 and to describe how the COVID-19 pandemic had affected their lives. Each participant then read the parking lot scenario used in Study 4 and viewed the schematic photo of the parking lot corresponding to his or her condition (Fig 3). Next, participants were asked to rate their overall evaluations of the parking lot management on a 7-point scale from 1 (*low evaluation*) to 7 (*high evaluation*).

We then measured participants’ cognitive dissonance (e.g., despair, disappointment, anger, hostility and discomfort) on a 7-point scale from 1 (*not at all*) to 7 (*very much*). These items were adopted from the dissonance scale [35] and were averaged into a single index of *dissonance* (α = .969). We also measured the extent to which participants’ attitudes toward the parking lot management were a function of self-attribution. For this purpose, we asked participants to rate their agreement with the following statements on a 7-point scale from 1 (*not at all*) to 7 (*very much*): “If I choose to park in this parking lot, it means that I like it,” “My feelings toward the parking experience reflect my attitude toward the parking lot management,” “My parking experience echoes my view of the parking lot management” and “If I love the parking lot management, it means that I am a loyal customer.” These items were adapted from the self-attribution scale [36] and were averaged into a single index of *self-attribution* (α = .87).

Finally, as a manipulation check of the physical constraint manipulation, participants rated the degree to which they felt they were captives on a scale from 1 (*very little*) to 7 (*very much*). A full description of the study can be found in the (S6 Study).

### 11.2 Results and discussion

#### Manipulation check of physical constraints

As expected, we found a significant effect of experimental condition on perceived captivity (*F*(1, 280) = 12.18, *p* < .001; η^*2*^ = .042). Specifically, participants in the constrained condition had a stronger sense of captivity (*M* = 3.38, *SD* = 2.03) than did participants in the unconstrained condition (*M* = 2.61, *SD* = 1.69). As in prior studies, the average rating of perceived captivity did not exceed mid-scale.

#### Main analysis

An ANOVA of participants’ overall evaluation of the parking lot management revealed a significant effect of experimental condition (*F*(1, 280) = 20.66, *p* < .001; η^*2*^ = .069). Specifically, as predicted in H1, participants in the constrained condition reported higher evaluations (*M* = 5.26, *SD* = 1.32) than did participants assigned to the unconstrained condition (*M* = 4.51, *SD* = 1.45).

#### Ruling out alternative accounts

An additional ANOVA with dissonance as the dependent variable yielded a non-significant effect (*F*(1, 280) = .58, *p* = .45; η^*2*^ = .002). Similar results were obtained when self-attribution was the dependent measure (*F*(1, 280) = .15, *p* = .69; η^*2*^ = .001). Moreover, when the dissonance and self-attribution indices were included as control variables in the main analysis, the effect of experimental condition remained significant (*F*(1, 280) = 25.91, *p* < .001; η^*2*^ = .085). Indeed, a parallel mediation analysis (Model 4, [32]) further emphasized that dissonance (*b* = .067, *SE* = .087, 95%, CI: -.103 to .242) and self-attribution (*b* = .024, *SE* = .062, 95%, CI: -.098 to .147) were not significant mediators of the positive effect of physical constraints.

Together, the results of Study 5 provide additional support to H1 and rule out the alternative accounts of cognitive dissonance and self-attribution.

## 12. Study 6: Reversing the positive effect of physical constraints

Studies 1–5 demonstrated the positive effects of physical constraints imposed on consumers in different settings. Recall that, in each of these studies, although constrained participants perceived the extent of their captivity as greater than that of unconstrained participants, they nevertheless rated these physical constraints as relatively mild (below mid-scale). Study 6 sought to test H3, which proposes that the positive effect of physical constraints is undermined when extreme constraints are imposed.

Like Study 2, this study investigated physical constraints in the context of student-university relationships. To avoid exposing participants to a potentially distressful situation, we used a scenario-based design. Specifically, we presented each participant with one of three scenarios describing different lecture settings in which the professor either keeps the class door open (no constraint), closes the class door (mild physical constraint), or locks the class door with a key (significant physical constraint). We expected to replicate the positive effect of physical constraints in the closed-door condition, but expected that the effect would be reversed in the locked-door condition (H3).

### 12.1 Method

Two hundred twenty-one undergraduate participants (49% women, *M*_age_ = 22.48) took part in this experiment for course credit. Participants were each randomly assigned to one of three conditions: open door, closed door, and locked door. All participants were asked to imagine that as part of a course they were taking at the university, a faculty member from the management faculty, who was not the regular course lecturer, had come to give an important guest lecture that was part of the course requirements. Then, participants were exposed to the manipulation corresponding to their conditions: Participants in the open-door condition were told that the guest lecturer kept the classroom door open. Participants in the closed-door condition were told that when the guest lecturer entered the classroom, he carefully closed the classroom doors, and informed the students in class that it was important for him that students not to "roam around the room" during the lecture. Participants in the locked-door condition were told that the lecturer locked the classroom door with a key and made the same statement. In addition, all participants were told that the guest lecturer did not complete the lecture on time and continued into the time of the break.

Participants were asked to rate their likelihood of being satisfied with this guest lecturer on a 7-point scale from 1 (*not likely*) to 7 (*very likely*) and the extent to which they would like the lecturer to give additional lectures on a 7-point scale from 1 (*not at all*) to 7 (*very much*). These two ratings were highly correlated (α = .86) and therefore were averaged into a single index of attitude toward the lecturer. Finally, participants rated the degree to which they felt they were captives of the lecturer on a 7-point scale from 1 (*very low*) to 7 (*very high*). A full description of the study can be found in the (S7 Study).

### 12.2 Results and discussion

#### Manipulation check of physical constraints

As expected, we found a significant effect of experimental condition on perceived captivity (*F*(2, 218) = 61.51, *p* < .001; η^*2*^ = .36). Specifically, participants who read the locked door scenario rated their sense of captivity higher (*M* = 5.26, *SD* = 1.62) than did participants who read the closed door scenario (*M* = 3.96, *SD* = 1.83, *p* < .001) or the open-door condition (*M* = 2.25, *SD* = 1.49, *p* < .001). The difference in perceived captivity between the closed- and open-door scenarios was also significant (*p* < .001). Importantly, perceived captivity in the locked-door condition was higher than mid-scale.

#### Main analysis

An ANOVA of attitude toward the guest lecturer as a function of experimental condition revealed a significant effect (*F*(2, 218) = 26.82, *p* < .001; η^*2*^ = .19). Specifically, in line with H1, participants in the closed-door condition rated their attitudes toward the lecturer more positively (*M* = 4.34, *SD* = 1.31) than did participants in the open-door condition (*M* = 3.93, *SD* = 1.34, *p* < .07). Participants in the locked-door condition rated the lecturer less favorably than did participants in the closed-door condition (*M* = 2.78, *SD* = 1.41, *p* < .001) and, importantly, and in line with H3, rated him less favorably than did participants in the open-door condition (*p* < .001). Indeed, participants in the locked-door condition rated the lecturer unfavorably and significantly below mid-scale (*p* < .001).

The results of this study show that the presence of physical constraints has a positive effect on consumers’ evaluations only when consumers perceive these constraints as mild. Under more extreme constraint perceptions, the effect is reversed, such that consumers perceive the provider as generally unfavorable and, specifically, less favorably than they do when they are not subject to physical constraints (H3).

## 13. General discussion

This research has shown that, contrary to the intuition that consumers generally object to restrictions on their freedom of movement, the presence of mild physical constraints may enhance consumers’ evaluations of a service provider (H1), as reflected in their reported attitudes towards the service provider (Studies 1, 3–6) or willingness to re-engage with the service provider (Study 2). We obtained evidence for this positive effect in six experiments across a variety of settings, and showed that the mere act of constraining consumers inherently provides some value. Indeed, we theorized and showed that this value stems from the fact that physical constraints provide consumers with a sense of structure (H2)—a sense that consumers generally value [e.g., 7]. We also ruled out alternative mechanisms such as self-attribution or efforts to reduce cognitive dissonance. Finally, we revealed that consumers respond positively to physical constraints only when these constraints are mild; the positive effect is reversed when consumers perceive the constraints as extreme (H3).

### 13.1 Theoretical contributions

Our findings contribute new evidence to the emerging understanding that consumers inherently value structure in their environment. Indeed, consumers’ desire for structure in consumption is known to drive product use [7], and several service providers have developed strategies that enhance structure in the consumption environment. In showing that the desire for structure leads consumers to embrace mild constraints on their freedom of movement, our research suggests that such constraints can serve as a unique source of utility for consumers and as a practical tool that service providers may use to strengthen their relationships with employees and consumers.

In our studies, we measured participants’ perceptions of being physically constrained on the basis of their perceptions of being held captive by the service provider. Participants’ positive responses to these feelings of captivity suggest that our effect may be considered a variant of the classic Stockholm syndrome, a well-known psychological phenomenon in which hostages express empathy for, and even trust in, their captors. [37–39] In classic captivity situations, captives are physically unable to escape their captors and thus may develop feelings of empathy as a coping mechanism [39]. In consumption contexts, in contrast, “captive” consumers are free to leave the service provider at any time—yet, as our findings suggest, embrace constraints on their freedom, as these provide a desired sense of structure.

### 13.2 Practical implications

Our findings have clear practical implications. Specifically, they suggest that service providers may benefit from creating conditions that restrict consumers’ freedom of movement. This finding—and, more broadly, the finding that consumers gain utility from structure in in their surrounding—is of high relevance in the modern world, where consumers face a deluge of information about products, services, and companies, and may gain utility from structured environments. Of importance, our results suggest that managers do not need to exert effort to justify their inclusion of physical constraints as part of the consumption experience, since the mere inclusion of these constraints enhances consumers’ sense of structure, regardless of whether such constraints seem to be directly related to fulfilling their goals.

The desire for structure may be further enhanced during the COVID-19 pandemic, when people across the world are encountering chaos and uncertainty. In one research [7] that studied consumers who experienced lack of control in their lives after the September 11^th^ terror attack (similar to what many of us have recently experienced due to the COVID-19 pandemic) noted that these consumers tended to seek structure in the consumption environment to regain a sense of control. Notably, the pandemic has not only introduced chaos but also severe restrictions on consumers’ movements, such as lockdown and social distancing, resulting in discomfort, unease, and even active resistance. Consumers are instructed where to stand in line in service establishments, where to sit in class, and the distance to maintain from others while exercising. In many cases, consumers chafe against such restrictions, perhaps perceiving them as overly extreme. The findings of the current research may suggest an opportunity for service providers to benefit from the need to impose such restrictions. If implemented in a manner in which they are not perceived as extreme, such physical restrictions may contribute not only towards safety and health but also towards a sense of structure that consumers might embrace amid the chaos of the pandemic.

### 13.3 Future research

Future research may consider constraints on consumers’ *virtual* movements in the digital sphere. For example, consumers may be directed to visit only a specific range of pages when browsing within a specific app, website, or social network, or directed to specific paths while using virtual reality technologies and navigating within the virtual environment. Likewise, forcing people to leave their cameras on when using videoconferencing tools (e.g., Zoom or Teams) might constrain their movements. Our findings (drawn from the physical domain) suggest that such constraints may enhance virtual experiences and individual’s evaluations of the firms that initiate those experiences.

More generally, it would be interesting to explore whether our findings regarding physical constraints may have broader implications for the numerous life situations in which consumers and employees are subjected to restrictive policies as a condition of participation. Some schools impose a dress code, which may enhance a sense of constraint or captivity, particularly when students compare themselves with fellow students from schools with more relaxed dress codes. At universities, some professors impose strict requirements on class attendance, assignment completion, or laptop use. As employees, we sometimes face restrictive rules at work. As consumers we are often faced with consumption situations that limit our freedom, such as menus offering limited options. Our findings point to the possibility—one that warrants further exploration—that such limitations might actually enhance people’s evaluation of the corresponding activities. Such a phenomenon would also have implications for regulatory policies aimed at preventing firms from imposing unnecessary restrictions on consumers. That is, it is possible that, in certain situations, an organization’s restrictive policies may align with consumers’ needs and even increase their satisfaction. It would also be of interest to explore the effects of specific service provider policies that consumers might tend to perceive negatively, and evaluate whether such policies might in fact, elicit positive evaluations when they are sufficiently mild.

## Supporting information

S1 Study Test for all studies. (DOCX)

S2 Study The positive effect of physical constraints on consumer evaluations. (DOCX)

S3 Study The positive effect of physical constraints on consumer choices. (DOCX)

S4 Study The mediating role of sense of structure. (DOCX)

S5 Study Process via moderation: Manipulating sense of structure. (DOCX)

S6 Study Ruling out alternative accounts. (DOCX)

S7 Study Reversing the positive effect of physical constraints. (DOCX)
