# Supplementary material for: The positive effect of physical constraints on consumer evaluations of service providers
Source: PLoS One. 2022 Oct 10;17(10):e0275348. doi: 10.1371/journal.pone.0275348 (PMC9550037; doi:10.1371/journal.pone.0275348)
Supplement: S1 Study — (DOCX) [file pone.0275348.s001.docx]

S1 Tests for All Studies

We conducted a set of tests (pre-registered *https://osf.io/fz8pr*) to test each of our experimental scenarios on the following aspects (1= not at all, 7 = very much): (a) the extent to which the described consumption experience is surprising, (b) the extent to which the described consumption experience is realistic, and (c) the degree to which participants can justify the service provider’s behavior.

|  |  | Not tied to chair | Tied to the chair |  |
| --- | --- | --- | --- | --- |
| Hairdresser setting  (Study 1) | Surprising | 2.62 (2.09)^x^ | 6.60 (.89)^y^ |  |
|  | Realistic | 4.85 (1.72)^a^ | 1.48 (1.05)^b^ |  |
|  | Justifiable | 5.19 (1.99)^a^ | 1.44 (.84)^b^ |  |
| n = 102, 50% women, *M*_age_ = 25.02 | | |  |  |
|  |  | Open door | Closed door | Baseline |
| Lab door setting  (Study 2) | Surprising | 3.27 (2.05)^g^ | 5.68 (1.38)^h^ | 2.55 (2.21)^l^ |
|  | Realistic | 4.54 (1.81)^s^ | 2.38 (1.56)^l^ | 5.41 (1.49)^h^ |
|  | Justifiable | 4.90 (1.68)^s^ | 2.64 (1.44)^l^ | 5.43 (1.52)^h^ |
| n = 151, 49% women, *M*_age_ = 25.28 | | |  |  |
|  |  | No marking | Permitted flow was marked |  |
| Parking lot setting (Studies 3-5) | Surprising | 2.29 (1.90)^c^ | 2.54 (1.44)^c^ |  |
|  | Realistic | 4.87 (2.27)^h^ | 5.10 (1.75)^h^ |  |
|  | Justifiable | 5.21 (2.06)^h^ | 5.42 (1.70)^h^ |  |
| n = 102, 50% women, *M*_age_ = 25.02 | |  |  |  |
|  |  | Open door | Closed door | Locked door |
| Class door setting  (Study 6) | Surprising | 3.90 (2.07)^o^ | 3.22 (2.02)^t^ | 5.43 (1.54)^n^ |
|  | Realistic | 3.90 (1.83)^o^ | 5.12 (1.49)^n^ | 2.63 (1.87)^k^ |
|  | Justifiable | 4.84 (1.93)^v^ | 4.76 (1.82)^v^ | 2.73 (1.60)^k^ |

n = 151, 49% women, *M*_age_ = 25.28

Note: Cells in each pre-test with unlike subscripts differ at *p* < .05.
