# Supplementary material for: The positive effect of physical constraints on consumer evaluations of service providers
Source: PLoS One. 2022 Oct 10;17(10):e0275348. doi: 10.1371/journal.pone.0275348 (PMC9550037; doi:10.1371/journal.pone.0275348)
Supplement: S2 Study — (DOCX) [file pone.0275348.s002.docx]

S2 Study 1 – The Positive Effect of Physical Constraints on Consumer Evaluations

**Sample**: *n* = 56, 52% female, *M*_age_ = 27. Participants received a haircut/blow-dry, paid $5 for the service, and completed a short questionnaire (presented below).

**Procedure and Questionnaire:** All participants were invited to receive a haircut / blow-dry. Participants were each randomly assigned to either a constrained condition (in which they would be tied to a chair with a belt) or a non-constrained condition (in which they would sit freely). Participants in each condition were not aware of the other condition (e.g., participants in the non-constrained condition were not aware of the constrained condition and did not see the belt used in that condition).

*Constrained condition:* The hairdresser used a belt to tie each participant to the chair and told them that this is how they would receive their haircut.

*Non-constrained condition:* Participants sat freely on the chair.

(see photos in Figure 1).

*Both conditions:* After receiving their haircut/blow-dry, all participants completed the following one-page questionnaire.

*Dependent measure*

To what extent do you like the haircut/blow-dry that you received?

| Not at all |  |  |  |  |  | Very much |
| --- | --- | --- | --- | --- | --- | --- |
| 1 | 2 | 3 | 4 | 5 | 6 | 7 |

How satisfied are you with your haircut/blow-dry?

| Very unsatisfied |  |  |  |  |  | Very satisfied |
| --- | --- | --- | --- | --- | --- | --- |
| 1 | 2 | 3 | 4 | 5 | 6 | 7 |

Would you like to return and receive another haircut/blow-dry, if that were possible?

| Not at all |  |  |  |  |  | Very much |
| --- | --- | --- | --- | --- | --- | --- |
| 1 | 2 | 3 | 4 | 5 | 6 | 7 |

Would you recommend participating in this research to others?

| Not at all |  |  |  |  |  | Very much |
| --- | --- | --- | --- | --- | --- | --- |
| 1 | 2 | 3 | 4 | 5 | 6 | 7 |

*Manipulation check item*

Did you feel that you were a captive of the hairdresser?

| Not at all |  |  |  |  |  | Very much |
| --- | --- | --- | --- | --- | --- | --- |
| 1 | 2 | 3 | 4 | 5 | 6 | 7 |

To conclude, please complete a few background details for research purposes only:

Gender -

- Male
- Female

Age: ____ years

Mother language: ___
