# Supplementary material for: The positive effect of physical constraints on consumer evaluations of service providers
Source: PLoS One. 2022 Oct 10;17(10):e0275348. doi: 10.1371/journal.pone.0275348 (PMC9550037; doi:10.1371/journal.pone.0275348)
Supplement: S3 Study — (DOCX) [file pone.0275348.s003.docx]

# S3 Study 2 - The Positive Effect of Physical Constraints on Consumer Choices

**Sample:** *n* = 154, 47% female, *M*_age_ = 23.21. Participants were undergraduate students who completed the experiment in return for course credit.

**Procedure and Questionnaire:** In this study we used the door of the behavior research lab to manipulate participants' perceptions of being physically constrained. Upon entering the lab, each participant was randomly assigned to one of three conditions: a *closed-door* condition, an *open-door* condition, or a *control* condition.

*Closed-door condition:* The experimenter told the participants that the lab door must remain closed during the experiment, and participants would not be permitted to leave the lab. She then closed the door and sat near the closed door.

*Open-door condition:* The experimenter told participants that the lab door would remain open during the experiment and participants were permitted to leave the lab as they wished. She then opened the door and sat at a distance from the door.

*Control condition*: The door of the lab was closed as it usually would be during a lab experiment. The experimenter made no reference to the door in her instructions, and sat in her regular place (near the experimenter’s table).

*All conditions*: First, participants completed a filler task, and were then told about an experimental panel that the behavioral lab researchers use when conducting their studies, whose members receive payment for their participation in experiments (in contrast to the current participants, who were receiving course credit). Participants were told that the researchers who manage this panel were recruiting new panel members. Participants were requested to choose whether they would like to take part in the paid panel and to state the number of studies they would be willing to complete in the paid panel. Finally, participants completed the questionnaire below.

For clarity of presentation, the text below includes a title for each page. In the experiments participants did not see these titles.

*Page 1: Filler task*

Participants were asked to complete a brand-name recognition task. All participants were given 9 “jumbled” names of well-known brands (e.g., TOFSOMCIR) and were asked to identify the brands (Microsoft).

| **C D A L M N S N D O** |
| --- |
| **R T L E S G L G I S E E N O T** |
| **S O O G M N L I B D A** |
| **H P Z I T Z U A** |
| **S T I F I F A E I N** |
| **A M E L I A R N S I A R N I E C** |
| **S K T A C U R B S** |
| **G B E U N R G I K R** |
| **I E N K E H E N** |

*Page 2: Dependent measure*

Would you like to take part in the paid panel?

- Yes
- No

*Page 3: Manipulation check item*

Please rate the degree to which you feel that you are a captive of the experimenter (1 *= not at all*, 7 *= very much*).

*Page 4*: *Demographics*

The following background questions refer to you.

Gender

- Male
- Female

Age: ____ years
