# Supplementary material for: The positive effect of physical constraints on consumer evaluations of service providers
Source: PLoS One. 2022 Oct 10;17(10):e0275348. doi: 10.1371/journal.pone.0275348 (PMC9550037; doi:10.1371/journal.pone.0275348)
Supplement: S4 Study — (DOCX) [file pone.0275348.s004.docx]

# S4 Study 3 - The Mediating Role of Sense of Structure

**Sample**: *n* = 256, 52% female, *M*_age_ = 23.58. Participants were recruited to participate in an online study for course credit.

**Procedure and Questionnaire:** Participants were asked to imagine that they were about to leave the parking lot where they regularly parked, right after they were in the gym. Next, participants were each randomly assigned to one of three conditions:

*Unconstrained condition*: We asked participants to imagine driving through the parking lot in any direction they desire, including through empty parking spaces (as schematically presented in Figure 2a in the paper).

*‘Constrained with a short path’ condition*: We asked participants to imagine that the parking lot’s management marked the permitted directional flow on the parking lot, and that driving in the opposite direction or through empty places was not permitted. Specifically, based on their marked parking spot, their way out of the parking lot was the shortest possible path (as schematically presented in Figure 2b in the paper)

*‘Constrained with a long path’ condition*: We told the same story as in the useful-constraint condition. However, we marked the arrows flow in the parking lot in the opposite direction, such that their path out of the parking lot was much longer (as schematically presented in Figure 2c in the paper)

All conditions: All participants completed the following questionnaire. For clarity of presentation, the text below includes a title for each page. In the experiments participants did not see these titles.

*Page 1: Scenario*

Imagine that you have a membership for a gym that is within driving distance of your home. Hence you need to use your car every time you go to the gym. The gym is located in a high-rise building that includes a private four-level parking lot.

| ***Unconstrained condition*** | ***‘Constrained with a short path’ condition*** | ***‘Constrained with a long path’ condition*** |
| --- | --- | --- |
|  | Once you enter the parking lot you find that the parking lot management has marked the driving routes inside the parking lot, directing consumers to drive in the parking lot only in one direction.  You park your car in the parking space marked in red (as demonstrated in the figure). | |
| When you were about to leave the parking lot, you saw that you could move freely. For example, you could decide to go forward or backward. Also, if there are free crossings you can shorten the path and drive through parking spaces.  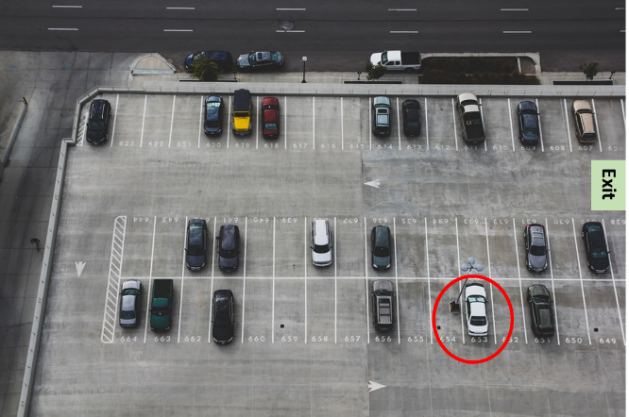 | When you were about to leave the parking lot, you look at the signs, and you understand that you can quickly and easily integrate with the direction of the arrows and get out of the parking lot from your parking space in the shortest way.  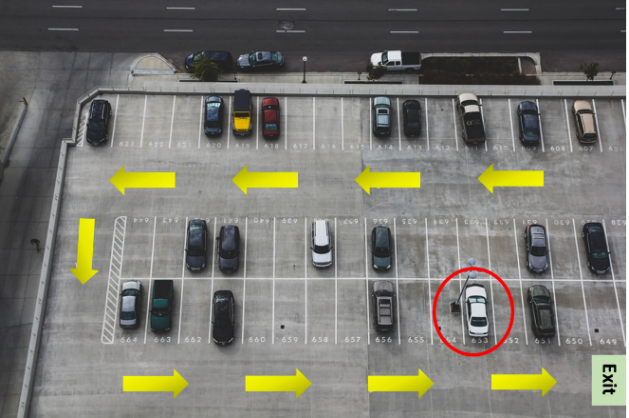 | When you were about to leave the parking lot, you look at the signs, and you understand that in order to exit the parking lot, you must drive backwards from your parking space and go around the parking lot, as you follow the arrows.  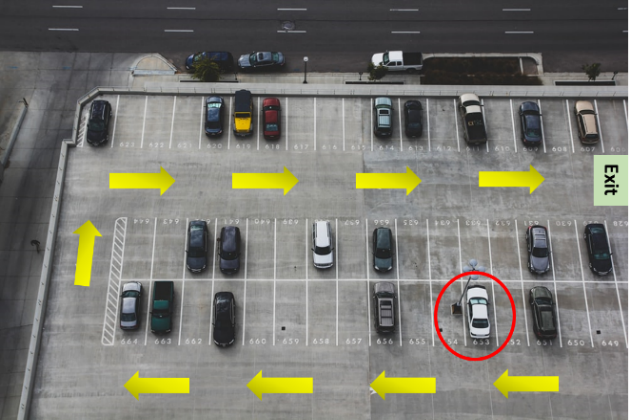 |

*Page 2:*

Please rate your overall evaluation of the parking lot management on a 7-point scale from 1 (*low evaluation*) to 7 (*high evaluation*)

| Low Evaluation |  |  |  |  |  | High Evaluation |
| --- | --- | --- | --- | --- | --- | --- |
| 1 | 2 | 3 | 4 | 5 | 6 | 7 |

*Page 3:*

Please rate the extent to which you believe the parking lot management establishes a structured consumption experience, on a 7-point scale from 1 (*not at all*) to 7 (*very much*).

| Not at all |  |  |  |  |  | Very much |
| --- | --- | --- | --- | --- | --- | --- |
| 1 | 2 | 3 | 4 | 5 | 6 | 7 |

*Page 4: Manipulation check*

Please rate the degree to which you feel as if you are a captive of the parking lot’s management.

| Not at all |  |  |  |  |  | Very much |
| --- | --- | --- | --- | --- | --- | --- |
| 1 | 2 | 3 | 4 | 5 | 6 | 7 |

*Page 5: Manipulation check*

To what extent do you feel that the parking lot management policy facilitates the way out of the parking lot?

| Not at all |  |  |  |  |  | Very much |
| --- | --- | --- | --- | --- | --- | --- |
| 1 | 2 | 3 | 4 | 5 | 6 | 7 |

*Page 6*: *Demographics*

The following background questions refer to you.

Gender

- Male
- Female

Age: ____ years
