# Supplementary material for: The positive effect of physical constraints on consumer evaluations of service providers
Source: PLoS One. 2022 Oct 10;17(10):e0275348. doi: 10.1371/journal.pone.0275348 (PMC9550037; doi:10.1371/journal.pone.0275348)
Supplement: S5 Study — (DOCX) [file pone.0275348.s005.docx]

# S5 Study 4 - Process via Moderation: Manipulating Sense of Structure

**Sample**: *n* = 280, 46% female, Mage = 35.02. Participants were recruited to participate in an online study for which they received $1 USD.

**Procedure and Questionnaire:** Participants were each randomly assigned to one of four conditions in a 2(structure perception: positive vs. negative) × 2(physical constraints: unconstrained vs. constrained) between-subjects design.

First, we manipulated structure perceptions. Participants in the positive-structure-perception condition were asked to think of situations in which order and structure had helped them to cope, and to give a specific example of such a situation. Participants in the negative-structure-perception condition were asked to write about an example in which order and structure had distracted them from coping effectively with a specific situation.

Then we presented participants with a parking lot scenario similar to that used in Study 3, accompanied by a schematic photo of the parking lot (see Figure 3 in the paper). In this study, the specific location of the participant’s car was not mentioned, and its location was not indicated in the photo. Participants in the unconstrained condition were asked to imagine going to their usual parking lot and driving through it in any direction they desired, including through empty places (as schematically presented in Figure 3a). Participants in the constrained condition were asked to imagine that the parking lot’s management had marked the permitted directional flow on the parking lot, and that driving in the opposite direction or through empty places was not permitted (as schematically presented in Figure 3b).

All participants completed the following questionnaire. For clarity of presentation, the text below includes a title for each page. In the experiments participants did not see these titles.

*Page 1: Order and Structure Perception Manipulation*

| *Benefits of order and structure* | *Downsides of order and structure* |
| --- | --- |
| Studies show that an orderly and structured lifestyle can help people cope with challenging situations. It is important for us to understand how you feel about this statement. Try to think of challenging situations in which order, organization and clear rules helped you deal with the situation.  For example, a sense of order and organization might come from having a regular routine, or situations where you know what to expect. Give us an example of such a situation, describe what is structured, organized and clear about it, and how it helped you cope with the challenge. | Studies show that an orderly and structured lifestyle can have a detrimental impact on people coping with various situations. It is important for us to understand where this insight meets you. Try to think of challenging situations in which order, organization and clear rules impaired your ability to deal with the situation.  For example, situations where you feel you have a consistent routine, or situations where you know what to expect. Give us an example of such a situation, describe what is structured, organized and clear in it, and how it negatively affected your ability to deal with the situation. |

*Page 2: Manipulation check*

Given the example of the situation you described, how much do you agree with each of the following statement:

I am in favor of having order and structure in my life

| Strongly disagree |  |  |  |  |  | Strongly agree |
| --- | --- | --- | --- | --- | --- | --- |
| 1 | 2 | 3 | 4 | 5 | 6 | 7 |

*Page 3: Scenario (cont.)*

Imagine that you have a membership for a gym that is within driving distance of your home. Hence you need to use your car every time you go to the gym. The gym is located in a high-rise building that includes a private four-level parking lot.

| ***Unconstrained condition*** | ***Constrained condition*** |
| --- | --- |
| Once you enter the parking lot you drive freely in all directions of traffic, so if you notice available parking spaces, you can take shortcuts (as shown in the picture below) until you find a parking space.  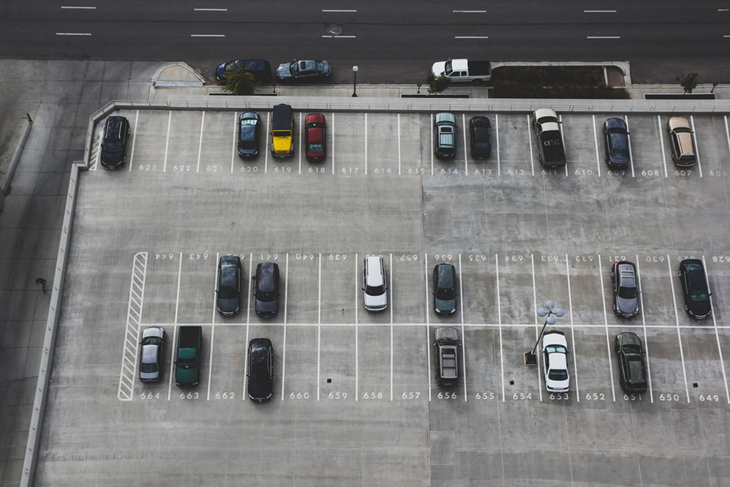 | Once you enter the parking lot you find that the parking lot management has marked the driving routes inside the parking lot, directing consumers to drive in the parking lot only in one direction (as demonstrated in the following picture). This marking requires you to look for a parking space as you drive on the marked route, without being able to take shortcuts, even if there are vacant parking spaces.  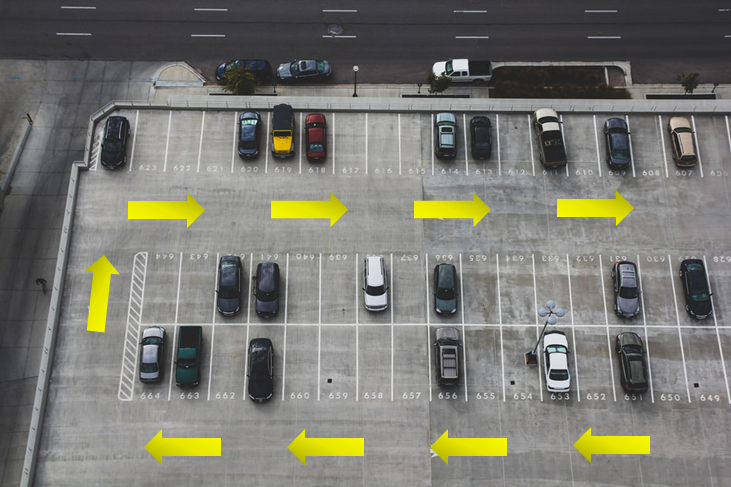 |

*Page 4:*

Please rate your overall evaluation of the parking lot management on a 7-point scale from 1 (*low evaluation*) to 7 (*high evaluation*)

| Low Evaluation |  |  |  |  |  | High Evaluation |
| --- | --- | --- | --- | --- | --- | --- |
| 1 | 2 | 3 | 4 | 5 | 6 | 7 |

*Page 5: Manipulation check*

Please rate the degree to which you feel as if you are a captive of the parking lot’s management.

| Not at all |  |  |  |  |  | Very much |
| --- | --- | --- | --- | --- | --- | --- |
| 1 | 2 | 3 | 4 | 5 | 6 | 7 |

*Page 6*: *Demographics*

The following background questions refer to you.

Gender

- Male
- Female

Age: ____ years
