# Supplementary material for: The positive effect of physical constraints on consumer evaluations of service providers
Source: PLoS One. 2022 Oct 10;17(10):e0275348. doi: 10.1371/journal.pone.0275348 (PMC9550037; doi:10.1371/journal.pone.0275348)
Supplement: S6 Study — (DOCX) [file pone.0275348.s006.docx]

# S6 Study 5 - Ruling Out Alternative Accounts

**Sample**: *n* = 282, 55% female, Mage = 35.88. Participants were recruited to participate in an online study for which they received $1 USD.

**Procedure and Questionnaire:** We collected data for this study during the outbreak of COVID-19. Participants were each randomly assigned to one of two conditions: unconstrained and constrained.

*Unconstrained condition:* We asked participants to imagine going to their usual parking lot and driving through it in any direction they desire, including through empty places (as schematically presented in Figure 3a in the paper).

*Constrained condition:* We asked participants to imagine that the parking lot’s management marked the permitted directional flow on the parking lot, and that driving in the opposite direction or through empty places was not permitted (as schematically presented in Figure 3b in the paper).

*Both conditions:* All participants completed the following questionnaire. For clarity of presentation, the text below includes a title for each page. In the experiments participants did not see these titles.

*Page 1:*

The recent period is characterized by many situations of uncertainty. For example, employment is unstable, it is not known whether restaurants will remain open, whether it will be possible to go to the beach, or how to plan the next vacation.

Please describe how COVID-19 has affected your life:

|  |
| --- |

*Page 2: Scenario*

Imagine that you have a membership for a gym that is within driving distance of your home. Hence you need to use your car every time you go to the gym. The gym is located in a high-rise building that includes a private four-level parking lot.

| *Unconstrained parking lot condition* | *Constrained parking lot condition* |
| --- | --- |
| Once you enter the parking lot you drive freely in all directions of traffic, so if you notice available parking spaces, you can take shortcuts (as shown in the picture below) until you find a parking space.  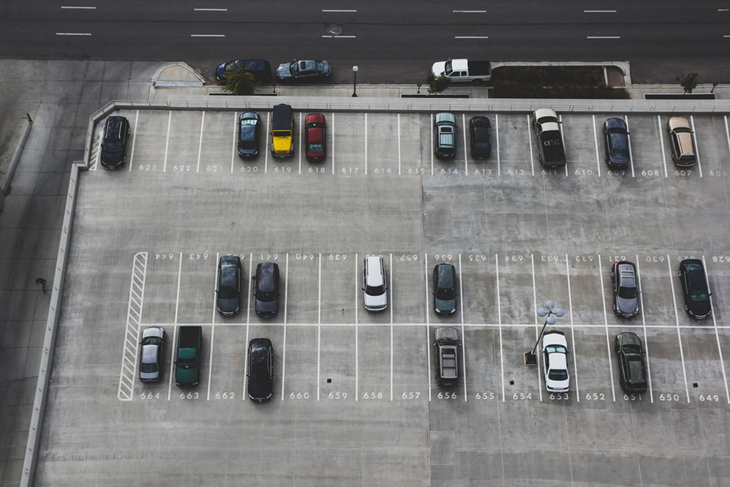 | Once you enter the parking lot you find that the parking lot management has marked the driving routes inside the parking lot, directing consumers to drive in the parking lot only in one direction (as demonstrated in the following picture). This marking requires you to look for a parking space as you drive on the marked route, without being able to take shortcuts, even if there are vacant parking spaces.  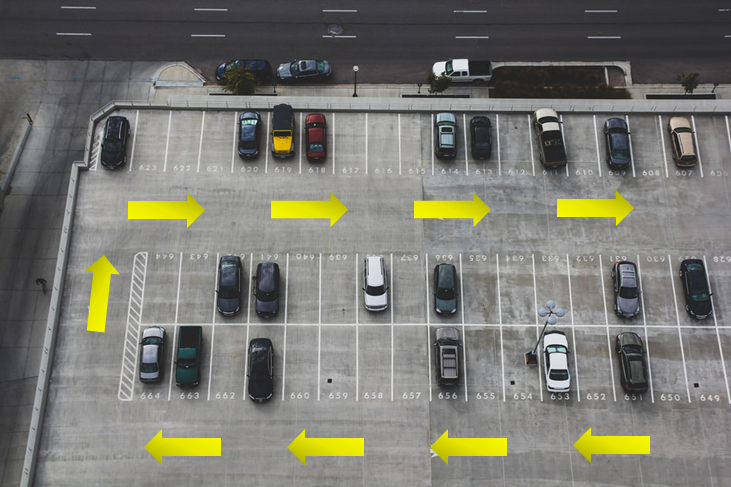 |

*Page 3: Dependent variable*

Please rate your overall evaluation of the parking lot management on a 7-point scale from 1 (*low evaluation*) to 7 (*high evaluation*)

| Low Evaluation |  |  |  |  |  | High Evaluation |
| --- | --- | --- | --- | --- | --- | --- |
| 1 | 2 | 3 | 4 | 5 | 6 | 7 |

*Page 4:*

Please mark to what extent your parking experience in the gym’s parking lot makes you feel (1 = *not at all*, 7 = *very much*) –

Despair

Disappointment

Anger

Hostility

Discomfort

Self-doubt: I wonder if I made the right decision.

I'm troubled by thoughts - maybe I should have entered a different parking lot.

*Page 5:*

Please mark the extent to which you agree with the following statements*:*

|  | Strongly disagree |  |  |  |  |  | Strongly agree |
| --- | --- | --- | --- | --- | --- | --- | --- |
|  | 1 | 2 | 3 | 4 | 5 | 6 | 7 |
| If I choose to park in this parking lot, that means that I like it. |  |  |  |  |  |  |  |
| My feelings toward the parking experience reflect my attitude toward the parking lot management. |  |  |  |  |  |  |  |
| My parking experience echoes my view of the parking lot management. |  |  |  |  |  |  |  |
| If I love the parking lot management, that means that I am a loyal customer. |  |  |  |  |  |  |  |

*Page 6*: *Manipulation check*

Please rate the degree to which you feel as if you are a captive of the parking lot’s management.

| Not at all |  |  |  |  |  | Very much |
| --- | --- | --- | --- | --- | --- | --- |
| 1 | 2 | 3 | 4 | 5 | 6 | 7 |

*Page 7*: *Demographics*

The following background questions refer to you.

Gender

- Male
- Female

Age: ____ years
