# Supplementary material for: The positive effect of physical constraints on consumer evaluations of service providers
Source: PLoS One. 2022 Oct 10;17(10):e0275348. doi: 10.1371/journal.pone.0275348 (PMC9550037; doi:10.1371/journal.pone.0275348)
Supplement: S7 Study — (DOCX) [file pone.0275348.s007.docx]

# S7 Study 6 - Reversing the Positive Effect of Physical Constraints

**Sample:** *n* = 221, 49% female, *M*_age_ = 22.48. Participants were undergraduate students who completed this online study in return for course credit.

**Procedure and Questionnaire:** All participants were asked to imagine that as part of a course they were taking at the university, a faculty member from the management faculty, who was not their regular course lecturer, would be giving an important guest lecture, and attendance was one of the course requirements. Then, participants were exposed to the manipulations corresponding to their conditions:

*Closed-door condition:* Participants were told that when the guest lecturer entered the classroom, he carefully closed the classroom doors, and informed the students in class that it was important for him that students not "roam around" during the lecture.

*Locked-door condition:* Participants were told that the lecturer locked the classroom door with a key and made the same statement as in the closed-door condition.

*Open-door condition:* Participants were told that the guest lecturer kept the classroom door open.

*All participants:* All participants were told that the guest lecturer did not complete the lecture on time and continued into the time of the break.

After reading their assigned scenarios, participants were asked to complete the following questionnaire. For clarity of presentation, the text below includes a title for each page. In the experiments participants did not see these titles.

*Page 1: Scenario*

Imagine that as part of a course you are taking at the university, a faculty member from the Faculty of Management, who is not the regular lecturer of the course, gave a guest lecture at one of the sessions. This is an important lecture, which is part of the study material in the course.

| *Open door condition* | *Closed door condition* | *Locked door condition* |
| --- | --- | --- |
| Note that when the lecturer entered the classroom, he left the classroom doors wide open, and announced that students were permitted to roam around the classroom during the lecture. Students could also leave the classroom (for example, to talk on the phone) and latecomers were permitted to enter the classroom during the lecture. | When the guest lecturer entered the classroom, he made sure to close the classroom doors and made it clear that it was important that students refrain from roaming around the classroom during the lecture. Students were not allowed to leave the classroom (for example, to talk on the phone) during the lecture, and  latecomers were not allowed to enter the classroom. | Note that when the lecturer entered the classroom, he made sure to lock the classroom doors with a key and made it clear that in this way he wants to make sure that students don’t “roam around the classroom” during the lecture. Students were not allowed to leave the classroom (for example, to talk on the phone) during the lecture, and  latecomers were not allowed to enter the classroom. |
| Also, the guest lecturer did not have time to finish the material within the lesson time and the lecture even spilled over into the break.  The lecture lasted about an hour and a half and at its conclusion, you were asked to provide feedback about the faculty member and the guest lecture he gave for the course. | | |

*Page 2: Dependent variables*

How would you have rated your satisfaction with the guest lecturer?

| Very dissatisfied |  |  |  |  |  | Very satisfied |
| --- | --- | --- | --- | --- | --- | --- |
| 1 | 2 | 3 | 4 | 5 | 6 | 7 |

If possible, would you like this guest lecturer to give an additional guest lecture in the future as part of the course?

| Not at all |  |  |  |  |  | Very much |
| --- | --- | --- | --- | --- | --- | --- |
| 1 | 2 | 3 | 4 | 5 | 6 | 7 |

*Page 3: Manipulation check*

When you imagine yourself attending the guest lecture described above, did you feel as if you were a captive of the guest lecturer?

| Not at all |  |  |  |  |  | Very much |
| --- | --- | --- | --- | --- | --- | --- |
| 1 | 2 | 3 | 4 | 5 | 6 | 7 |

*Page 4*: *Demographics*

The following background questions refer to you.

Gender

- Male
- Female

Age: ____ years
